# Therapeutic Potential of a Biodynamic Supplement on Skin Pressure Ulcers: A Randomized Clinical Study

**DOI:** 10.3390/biomedicines12081918

**Published:** 2024-08-22

**Authors:** Pasquale Ferorelli, Manfred Doepp, Stefano Lenzi, Roberto Rovelli, Gennaro Gisonna, Giuseppe Maierà, Francesco Antonelli, Massimo Radaelli, Anna Shevchenko, Giordana Feriotto, Carlo Mischiati, Ilaria Borromeo, Simone Beninati

**Affiliations:** 1 Saint George Campus—Scuola di Formazione Certificata Nazionale e Internazionale, Via Oprandi 1, 24065 Lovere, Italy; p.ferorelli@citozeatec.it (P.F.); gisonnag@hotmail.it (G.G.); maiera@polispecialisticopacini.it (G.M.); radaelli@saintgeorge.it (M.R.); 2 Department Psychology and Sports Science, Giessen Justus, Liebig University Gießen, 35398 Gießen, Germany; doepp@glessenjustus.dc; 3 Department of Health Engineering, Université Européenne de Bruxelles Jean Monnet, 1030 Brussels, Belgium; stefano.lenzi@yahoo.it; 4 Department of Agricultural and Environmental Sciences, University of Milan, 20133 Milan, Italy; rovelli@polytechnicmilan.it; 5 Scientific Association “A.R.S.S.—Associazione Ricerca Scientifica Sgurgola”, 00100 Rome, Italy; francescoantone@alice.it; 6 Department of Dermatology, Kabardine University, 121005 Nalchik, Russia; kabardineuniversity@dermatology.com; 7 Department of Chemical, Pharmaceutical and Agricultural Sciences, University of Ferrara, 44121 Ferrara, Italy; frn@unife.it; 8 Department of Neurosciences and Rehabilitation, University of Ferrara, 44121 Ferrara, Italy; msc@unife.it; 9 Department of Biology, University of Rome Tor Vergata, Via della Ricerca Scientifica, 00100 Rome, Italy; ilaria18scv@hotmail.it

**Keywords:** pressure ulcers, biodynamic therapy, nutritional supplement

## Abstract

Pressure ulcers (PUs) are a debilitating and often painful condition. They are localized lesions on the skin and/or underlying tissues and are common in the elderly, people with mobility difficulties, diabetics, and vascular disease or malnutrition, as well as in those requiring intensive or palliative care. The prevention and treatment of PUs involve strategies to optimize hydration, circulation, and nutrition. Nutrition plays a key role in pressure ulcer care because wounds require macronutrients and micronutrients to heal. Reports relating to the effectiveness of “Complementary Enzyme Therapy” also in the vulnological field led us to this study, the aim of which was to test the activity of a biodynamic food supplement (Citozym^®^) rich in carbohydrates, vitamins, and amylase and lactase and characterized by marked antioxidant activity. Citozym^®^ administered topically and/or systemically, and in particular in both administrations, in patients suffering from Pus, has shown a marked reduction in bedsores and, in many cases, complete healing. Furthermore, it was possible to observe a lower incidence of side effects compared to conventional therapies. The results obtained, confirmed by various tests and recognized by the scientific community, allow us to conclude that treatment with Citozym^®^ could represent a new and effective strategy for the treatment of PUs.

## 1. Introduction

Despite increasing attention over the past 20 years, the prevalence of PU has remained essentially unchanged while costs associated with treatment continue to increase [1]. Doctors and healthcare professionals can play a significant role in preventing PU by becoming aware of at-risk populations and implementing appropriate preventative strategies. Furthermore, doctors and healthcare professionals should be able to recognize early changes that occur before skin injury and correctly identify and classify PU to avoid delays in providing appropriate care. In individuals with normal mobility and mental state, prolonged pressure elicits a feedback response that requires a change in body position; however, when the feedback response is absent or impaired, sustained pressure ultimately leads to tissue ischemia, lesions, and necrosis [2]. Pathophysiology, risk factors, epidemiology, social and economic burdens, and the clinical presentation of PU are current topics in research projects [3]. Wound healing is a multicellular physiological process of the programmed and reproducible cooperation of the cells involved. It is one of the most complex biological processes that occur during human life. Wounds are the result of tissue breakdown with the loss of normal anatomical structure and consecutive loss of normal tissue function [4]. The wound-healing process generates tissue remaking and reconstitution as well as restoration of the tensile strength of damaged tissue. This begins within seconds of injury with homeostasis, followed by phases of inflammation, proliferation, and tissue remodeling to restore tissue integrity and the barrier function of the skin [5]. From a metabolic point of view, all these phases require an energy and protein substrate that is sufficient to ensure good wound healing. Ulcers with a poor healing prognosis include leg ulcers, such as venous, arterial, or mixed ulcers, PU, and neuropathic foot/leg ulcers [6]. PU, which affects a large number of individuals representative of the world’s population, represents a serious and growing threat with a negative economic impact. This pathology also leads to personal problems, both social and psychological. Inflicted wounds are difficult to heal and lead to repeated recurrences. At-risk patients require a thorough evaluation that includes a detailed medical history, skin examination, and evaluation of the patient’s support systems. Risk assessment tools have been developed to identify those at greatest risk and reduce the incidence of PU, with the idea that those at risk may benefit from more rigorous interventions [7]. There is a lack of agreement on predictive risk factors, which has led to the proliferation of various tools that include different variables of interest. Nutritional intake plays a key role in the repair of damaged tissues [8]. Recently, the patient’s diet has also been addressed as an adjuvant in the treatment of these dermatological pathologies [9]. In particular, it has been suggested that an adequate intake of macronutrients and micronutrients may play an important role in the healing process of ulcers. The proposed double-blind study presents data relating to the prospective, randomized, and controlled evaluation of the effects of a biodynamic nutritional supplement, which is particularly useful in this group of difficult-to-heal lesions and also for its ability to counteract enzymatic imbalances and for its documented antiseptic activities. Altered enzymatic machinery is a substantial biochemical feature of epidermal cell metabolism that changes the metabolic profile from oxidative phosphorylation to amplified glycolysis as well as increased lactate production under hypoxic conditions. The overexpression of several enzymes in PU has been indicated as a cause of delayed wound healing [10]. Therefore, being able to reprogram the function of these enzymes could represent an attractive avenue for the treatment of bedsores. Citozym^®^ is a product that belongs to a new generation of nutritional supplements, defined as “Biodynamic”, (i.e., active from a biochemical point of view), which can provide functional foods obtained from specific enzymatic conversions and, therefore, “nourish” the cells directly and quickly [11]. This experimental investigation aimed to use this nutritional supplement to verify its capacity as an authentic therapeutic target for the therapy of PU. The positive results obtained suggest that further investigations into biodynamic enzymology should focus on exploring the molecular mechanisms of various pathologies.

## 2. Materials and Methods

### 2.1. Patients

All participants selected from the hospital outpatient Department of the Investigative Dermatology Institute (IDI) Russia (Dr. A. Shevchenko) received verbal and written information about the study, and informed consent was obtained by everyone before the start of the experiment. Initially, 90 patients were included in the sample and selected, but 10 were withdrawn from the group due to personal characteristics and pathologies unrelated to the intervention. This study was conducted as a prospective, randomized, controlled, double-blind investigation to observe clinical parameters and patient experiences during therapy aimed at healing PU, comparing conventional treatment (see Supplementary Materials) with Citozym^®^ therapy. The researcher responsible for patient selection reviewed all patients and made the final decision on whether the patient met the inclusion criteria or not. Exclusion criteria from the study included patients with severe general diseases, patients who required local anesthesia for treatment, and patients who smoked. For the randomization procedure, an urn containing 80 numbers corresponding to the enrolled patients was prepared. The four sets of patients were divided as follows: “1–20 control”, “21 to 41 topical treatments”, “42 to 62 systemic treatments”, and “63 to 83 combined topical + systemic treatments”.

### 2.2. Fundamental Characteristics of the Sample Examined

**Figure 1:**
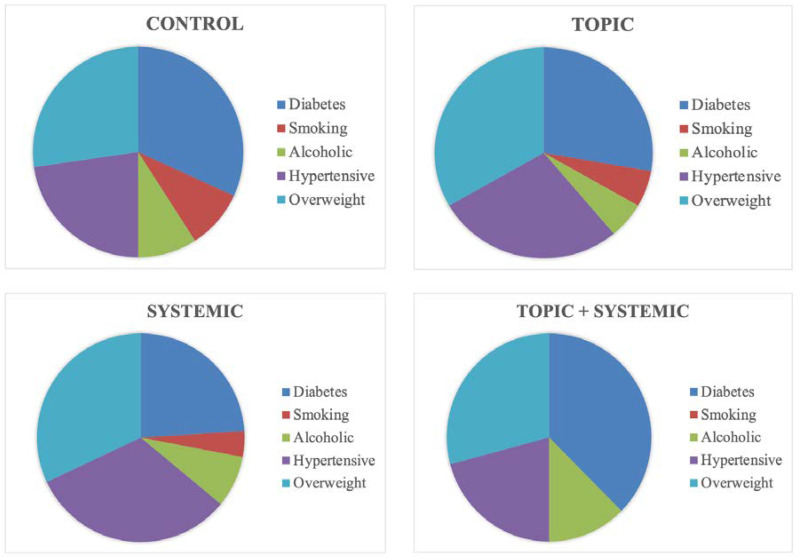
Presence of pathologies (diabetes, hypertension, and overweight) and dependencies (smoking and alcohol) related to the patients in the study groups (control, topic, systemic, and topic + systemic). Data are expressed as % over the total number of patients (*n* = 20) in each group.

To eliminate data influenced by events unrelated to the experimental protocol, the study required 3 months of preliminary observation. The implementation of the project and the observation of the results took 10 months (analysis and selection of patients; 4 months—staging of ulcers—1 month; experimentation—2 months; data analysis and healing potential—2 months; preparation of the project final report—1 month). All included subjects received a copy of the “Free and Informed Consent Form” and signed it after having been guided and informed on the procedures to be adopted during the study. All patients provided permission for this study, including the use of images (for recording metric data). All these supporting documents have been validated by an official ethics committee (UTVI Protocol 352-2022). The experimentation is part of the research project “Activation of a collaboration and scientific research program between the Department of Biology (M.S.M.F.N.) and Citozeatec s.r.l. (Food Supplements and Cellular Pathologies) RM 070622”. The selected patients underwent an initial evaluation and were monitored several times a week for 3 consecutive months. The observations, photographs, and measurements were carried out in the first two months of treatment. The third month allowed us to verify the permanence of the healing to avoid relapses of the pathology. The wounds of all patients were documented by measurements and photographs according to a sequence of wound bed preparation and dressings. The sample was composed of 80 men, divided into four groups of 20 patients (control, topic, systemic, and topic + systemic), and participants were selected on the basis of the following characteristics: overweight, hypertensive, smokers, consumes alcohol and diabetic (Figure 1).

Initial ulcer characteristics, lesion duration, lesion area, Pressure Ulcer Scale for Healing score (PUSH Tool 3) [12], and quality of life (QL) [13] varied considerably. The lesions ranged from small to large and lasted for months, showing various healing conditions. The primary objective was to calculate the percent rate of wound contraction using the re-epithelialization index (RI) over a two-month period. Secondary objectives assessed the healing rate based on the wound closure (millimeters per week of growth at wound edges), nutritional and laboratory parameters, and anthropometric measurements. Body weight and height were measured using a portable electronic scale. Since obesity contributes to immobility and subsequent pressure on skin surfaces, knowledge of the relationship between obesity and the development of pressure ulcers in intensive care patients could provide a better understanding of which patients are at high risk for pressure ulcers and allow more efficient prevention [14]. Nutritional intake was assessed before and after Citozym^®^ treatments. The body mass index (BMI) was calculated by dividing weight by height squared, expressed in kg/m^2^. All these parametric assessments were classified according to the guidelines suggested by CONSORT-Outcomes 2022 [15]. These types of evaluations made it possible to study a homogeneous group of volunteers (Table 1, Table 2, Table 3, Table 4 and Table 5).

### 2.3. Metric Measurements and Photographic Monitoring

The principal medical researchers measured and photographed all wounds weekly, always with the same photographic equipment (Huawei P30 Leica Quad Camera, Shanghai, China). A sterile ruler, a 125 MEB-6/150 vernier caliper (https://www.classichandtools.com/starrett-125-meb-6150-vernier-caliper/p1551?srsltid=AfmBOorHK0I1Bv91EcxqbsGsbnCEVOv4P3rqnOksI8DHo6JZvzqc3wKj (accessed on 20 August 2024)), and image processing software for surface area and perimeter calculations were used to collect and record wound measurement data. Many patients did not consent to the disclosure of images taken of the wounds being treated. The photographic images taken of patients were considered private data and treated as such. The classification of the observed ulcers was carried out in accordance with international standards, which included the following: Stage I: Intact skin with localized, non-blanchable erythema over a bony prominence. The area may be painful, hard, or soft and warmer or colder than the surrounding tissue. Darkly pigmented skin may not show visible whitening; however, the color of the Stage I ulcer will appear different from the color of the surrounding skin and indicates that the patient is at risk of further tissue damage if the pressure is not relieved. Stage II: A partial thickness wound presenting as a shallow, open ulcer with a red/pink wound bed. It may also present as an intact or open/ruptured blister filled with serum or sero-sanguineous fluid. Fragments of scaly skin may be present, which do not hide the depth of tissue loss. Stage III: Full-thickness wound. Subcutaneous tissue may be visible, but bones, tendons, and muscles are not exposed. Scaling or eschar may be present, but this does not mask the depth of tissue loss. Stage IV: A full-thickness wound with exposed bone, tendon, or muscle. Scaling or sloughing may be present in some parts of the wound bed, but this does not hide the depth of tissue loss.

### 2.4. Percentage Rate of Wound Contraction and Calculation of Linear Growth (Re-Epithelialization)

In addition to the metric data, it was also possible to calculate the percentage rate of contraction and the linear growth of the wound edges. The percentage rate of wound contraction could be used to monitor an ulcer over time. Weekly comparisons of wound area reduction rates can be converted into wound-healing trajectory curves, particularly in the first 2–4 weeks of treatment. Wounds that show a rate of area reduction of 10–15% per week predict healing; those showing 30–50% area reduction in the first 2–4 weeks have high healing potential.

### 2.5. Citozym^®^ Treatment Protocol

The administration of the nutritional supplement Citozym^®^ (Citozeatec Italia-FDA registration 12932524008 Pin no. bfJ3h263) was carried out according to a scheme considered useful for testing both the direct action of the product on the wound (topical effect) and the action exerted by oral administration (systemic), or both simultaneously (topical plus systemic) (see Supplementary Material).The possibility of being able to test the general improvement of the patient following the treatment adopted was conducted through investigation methods accepted by the scientific community [15]. Before treating a skin lesion, it is important to observe and evaluate it. For this assessment to be as objective and reproducible as possible, it is advisable to use internationally recognized classification tools. The staging of a lesion is important in order to use a common language that allows the different operators to be able to understand the lesion and together decide on therapeutic strategies by optimizing resources. There are different anatomical classifications; the most used are the EPUAP, 1997 (European Pressure Ulcer Advisory Panel, Guidelines on the Treatment of PU) and the NPUAP (National Pressure Ulcer Advisory Panel) [16,17]. The anatomical classification allows the depth of the wound to be identified, describing its progressive worsening. In these terms, it takes on an important prognostic meaning: the first two phases lead to an intact return, while in the subsequent ones, since the musculoskeletal system is involved, sequelae are expected, which require greater use of resources. The individual variables considered were as follows: an evaluation of wound surface reduction after treatment; re-epithelialization; the PUSH score; and quality of life (QV).

### 2.6. Re-Epithelialization

Keratinocytes, the main cellular component of the epidermis, are not only important for maintaining the barrier but also for its restoration in case of injury through a process known as epithelialization [18]. Epithelialization is defined as the process of covering the denuded epithelial surface [19]. The cellular and molecular processes involved in the initiation, maintenance, and completion of epithelialization are essential for successful wound closure. Many research efforts have focused on understanding these processes in both acute and chronic wounds. Calorie and protein requirements in healing wounds are higher than the average for non-wounded tissue. A poor diet causes a nutrient deficit and protein degradation and slows tissue recovery. To close the defect in the epidermis, keratinocytes at the wound edge must first loosen their adhesion to each other and to the basal lamina and must develop the flexibility necessary to support migration onto the newly deposited matrix. This process is modulated sequentially, starting from the disassembly of cell–cell, and cell–substrate contacts maintained through desmosomes and hemidesmosomes, respectively. Epithelialization plays a crucial role in wound healing as it is impossible to achieve wound closure if it fails. This phase of wound repair involves the initiation, proliferation, migration, and differentiation of keratinocytes at injury sites, along with the repair and reorganization of compromised dermal structures. The size, severity, and location of a wound can determine how quickly complete healing can be achieved. Generally, larger wounds take longer to heal than smaller wounds.

### 2.7. The Push Tool Test 3

This is a dynamic monitoring system in which the pressure ulcer is observed and evaluated, which classifies the lesion taking into account the surface, exudates, and the type of tissue involved [12]. The sub-scores for each of these characteristics can be recorded; then, the sub-scores are added to obtain the total score. Therefore, the total score measured over a period of time indicates whether the lesion has improved or not. The scale collects quantitative and qualitative characteristics useful for describing the lesion in the most objective way possible and allows the results of treatment to be compared over time. The length-by-width data are obtained from the estimate of the surface area in cm^2^. An evaluation of exudates is performed after dressing and before applying any topical agent to the wound. The estimate of exudates (drainage) is classified as absent, light, moderate, or strong. The tissue type refers to the types of tissue present in the wound bed; it is necrotic with a score of 4; squamous with a score of 3; if the wound is clean with granulation tissue, it is a score of 2; and epithelial tissue has a score of 1, while the score is 0 if the lesion is closed.

### 2.8. Quality of Life (QL)

The World Health Organization Quality of Life (WHOQOL) defines “Quality of Life” as an individual’s perception of their position in life in the context of the culture and value systems in which they live and concerning their goals, expectations, standards, and concerns [20]. In research, QL measurements can help determine whether new clinical treatments are effective enough in improving a patient’s life. This form of measurement is sometimes called the “patient-reported outcome”. Researchers need to establish the effects that a new treatment has on a subject. There is currently a broad consensus on some fundamental aspects that any tool aimed at quantifying such aspects must necessarily consider the following: physical functioning and well-being; psychological functioning and well-being (mainly emotional and cognitive aspects); social functioning and well-being; and physical symptoms (both those relating to the specific pathology and those resulting from any treatments for that pathology). Various parameters allow the evaluation of QL in patients treated with therapies that have the aim of improving patients’ state of health and potentially improving the patient’s QL. The QL parameters assessed are pain and discomfort, energy and effort, daily life, sleep and rest, mobility and working capacity. The values for each parameter are: 0 = very bad; 1 = tolerable; 2 = moderate; 3 = good; and 4 = excellent.

### 2.9. Statistical Analysis

The data obtained in this study were described as means, standard deviations (SDs), and standard errors or frequencies and percentages. The comparison between therapies and groups took into account the analysis of variance (ANOVA) model with two sources of variation. The comparison of two evaluation moments with respect to the quantitative variables was carried out with Student’s *t*-test for paired samples. Regarding wound-related variables, considering the possibility of patients with more than one ulcer, each patient was considered as a cluster. For the comparisons of interest, the estimation model for relationships with quantitative variables and the estimation model for relationships with clusters were used for dichotomous variables. *p* values < 0.05 indicated statistical significance. Data were analyzed with Stata/SEv.14.1 statistical software (Stata Corp LP, College Station, TX, USA).

## 3. Results and Discussion

### 3.1. Positive Effect of Cytozym^®^ on Re-Epithelialization Process

**Figure 2:**
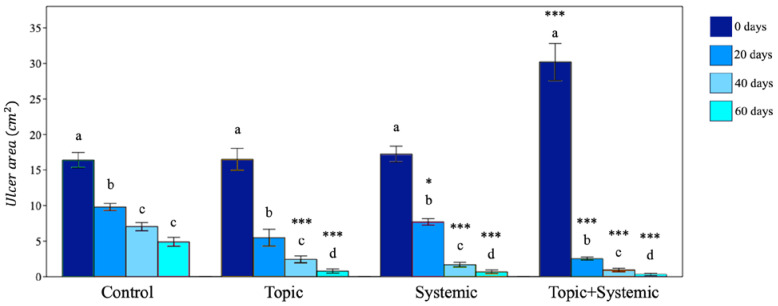
Variation in wound contraction during the planned treatments. The reduction in the wound area (cm^2^) indicates the speed of re-epithelialization of the wound. Data are expressed as mean ± SD (*n* = 20). One-way analysis of variance (ANOVA) was performed with Past 4.15. Significant differences within the same group (*p* < 0.05; ANOVA and Tukey–Kramer test) are reported with different letters in the column. All analyses were considered significant at *p* < 0.05 within each treatment group. When comparing the control to the topic, systemic, and topic+systemic groups, the significance was *** *p* < 0.001; * *p* < 0.05.

**Figure 3:**
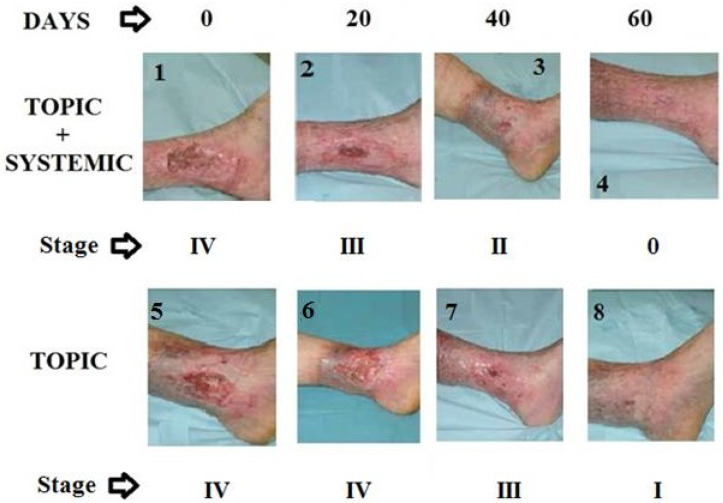
Representative photographic images of a reduction in PU in a 77-year-old patient treated with Citozym^®^ using the topical/systemic combination (1–4) and in a 67-year-old patient treated with topical therapy alone (5–8). Note the incomplete reduction in the wound in the exclusively topical treatment (photo 8) and the complete closure in the combined topical + systemic treatment (photo 4).

**Figure 4:**
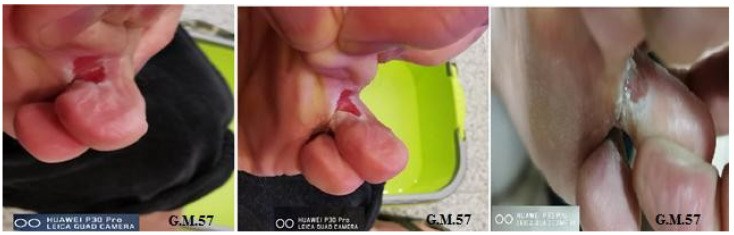
The reduction in the ulcer area treated with combined therapy for 60 days. Representative photographic images of pressure ulcer reduction in a 57-year-old diabetic patient treated with Citozym^®^ using the topical/systemic combination. (Note the more complete partial but sterile closure in the combined topical + systemic treatment).

**Figure 5:**
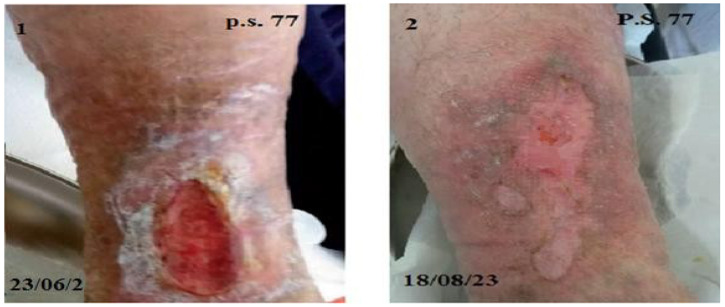
Reduction in pressure ulcer area treated with combined therapy for approximately 60 days. Representative photographic images of the reduction in PU in a 77-year-old diabetic patient treated with Citozym^®^, using the topical/systemic combination. The wound was reduced by approximately 75%.

**Figure 6:**
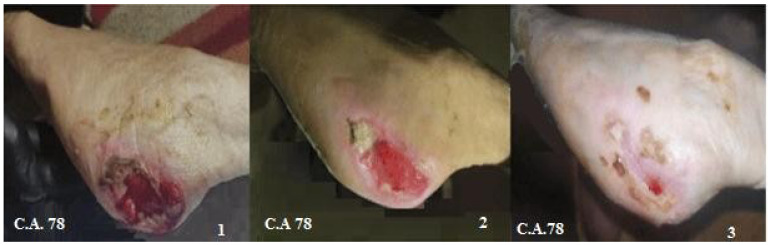
Reduction in the area of PU in the foot treated with combined therapy for 60 days. Representative photographic images of pressure ulcer reduction in a 78-year-old patient treated with Citozym^®^, using the topical/systemic combination. The wound was reduced by approximately 85%.

The decrease in the area of PU indicates the formation of epithelium covering the underlying connective tissue. Re-epithelialization describes the reemergence of a wound with new epithelium. The cellular and molecular processes involved in the initiation, maintenance, and completion of epithelialization are essential for successful wound closure. It is, therefore, a precious prognostic sign if there are reductions in the ulcer [21]. The controlled treatment of PU was carried out according to the last update of the WHS guidelines [22]. In the panel of Figure 2, it is evident that ulcers treated for 60 days with the topical or systemic procedure compared to the controls showed about 75% reduction in the wound area. The combined topical and systemic treatment reduced the wound area by 95%. The control treatment reduced the ulcer area by 25% over 60 days. The results presented in Figure 2, relating to the combined topical and systemic treatments, highlight the regenerative action of Citozym^®^ with regard to the formation of new epithelium, which is expressed by promoting wound closure and stimulating the growth of new tissues. These data also seem to be confirmed by the direct observation of the wound on the patient (Figure 3), where wound closure is highlighted more completely in the combined treatment compared to the topical treatment alone (Figure 4, Figure 5 and Figure 6).

In diabetic patients, hyperglycemia impairs neutrophil function and reduces host defenses [23]. This result leads to a tendency for bacterial infections that slow down and reduce wound healing. Patients with one or more of these risk factors accelerate the development of wounds that may be slow to heal and predispose to secondary infections. Without treatment, this type of wound can become infected. This can lead to extreme outcomes such as sepsis. The arrest of the tissue repair process of a lesion, linked to an increase in bacterial load, determines the appearance of the first and local signs of inflammation, which can be considered as the beginning of an evolving infection. The application of Citozym^®^ according to the protocol reported in the “Methods Section” has made it possible, in many cases, to control and reduce the increase in germs, allowing the tissue repair process to resume in an almost sterile environment.

### 3.2. PUSH Score

**Figure 7:**
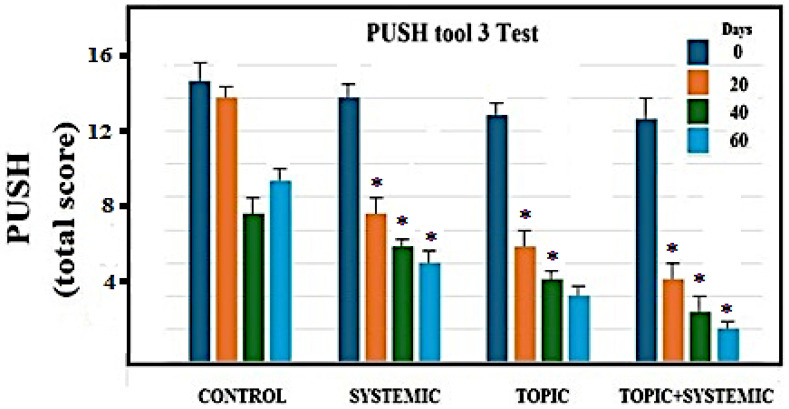
PUSH test tool derived from the collection of parameters indicated on patients examined and treated as described in the Methods section. The length-by-width data were obtained from the estimate of the surface area in cm^2^. The evaluation of exudates was performed after dressing and before applying any topical agent to the wound. Asterisks indicate significant values with respect to control untreated ulcers (*p* < 0.05).

Before treating a skin lesion, it is important to observe and evaluate it. For this assessment to be as objective and reproducible as possible, it was advisable to use internationally recognized classification tools. When staging a lesion, it is important to use a common language that allows the different operators to understand the lesion and decide on therapeutic strategies together, optimizing resources. The anatomical classification allows the depth of the wound to be identified, describing its progressive worsening. In these terms, it takes on an important prognostic meaning: the first two phases lead to an intact return, while in the subsequent ones, since the musculoskeletal system is involved, sequelae are expected, which require a greater use of resources. In addition to assessing the depth of the lesion, staging allows a prognostic measure in terms of time/healing. A stage III-IV lesion can be repaired over a very long time and with a much higher commitment of human and economic resources compared to a stage I-II lesion on which a timely and precise intervention can lead to resolution in a few days. Push Tool 3 is a dynamic monitoring system in which wound pressure is observed and measured. This classifies the lesion, taking into account the surface, exudates, and the type of tissue involved. Length-by-width data are obtained from the estimate of the surface area in cm^2^. An evaluation of exudates is performed after dressing and before applying any topical agent to the wound. A sub-score is recorded for each of these characteristics; then, the sub-scores are added to obtain the total score. Therefore, the total score measured over a period of time indicates whether the lesion has improved or not. Figure 7 highlights the results obtained with the systemic, topical, and combined topical plus systemic treatment, respectively. It appears clear that the closure of the wound following the re-epithelialization of the affected area occurred within the times foreseen by the trial and more rapidly in the combined treatment.

From Figure 7, it can be observed that the total score of the PUSH test in control patients was reduced by 36.17% over 60 days, while for the systemic treatment in the same period, it was reduced by 61.36%. Topical treatment reduced the total PUSH score by 73.1%. The combined treatment was more effective, bringing the total PUSH score to a value of 85%.

### 3.3. Correlation between Cytozym^®^ Use and Patients’ Quality of Life

Interest in the quality of life (QL) of patients has grown in recent times. The attention of healthcare personnel has shifted from therapy to the desire to understand the impact of healthcare treatments on the patient’s life. PUs are known to worsen patients’ quality of life on a psychological level. Patients’ lives are severely affected as they are completely dependent on their supportive environment and healthcare services [24]. QL is an important endpoint in medical and healthcare research, and QL research involves a variety of patient groups and different research projects. Various parameters allow the evaluation of QL in patients treated with therapies that aim to improve their state of health and potentially improve the patient’s QL. The QL parameters evaluated are pain and discomfort, energy and effort, daily life, sleep and rest, mobility, and work capacity. QL is an important concept in the field of health and medicine. QL is a complex concept that is interpreted and defined differently within and across disciplines, including health and medicine. Pressure ulcers have a significant effect on patients’ QL. For the present study, a questionnaire was proposed to all participants. The majority of subjects enrolled for the treatment reported psychosocial disorders, such as depression (60.5%), a negative perception of body image (67.8%), difficulties in relationships with peers (81.9%), physical and mental tiredness (75.7%), weakness during leisure activities (65.2%) and reduced physical activity (69.5%). A further questionnaire highlighted the presence of physical effects resulting from recurrent infections of ulcers or injuries to the lower limbs, which required hospitalization. Exuding ulcers was particularly problematic in its management because patients had to use additional materials, which is a process that is time-consuming and requires significant financial costs. Therefore, the physical, psychological, economic, and social consequences experienced by these patients significantly reduce their QL.

**Figure 8:**
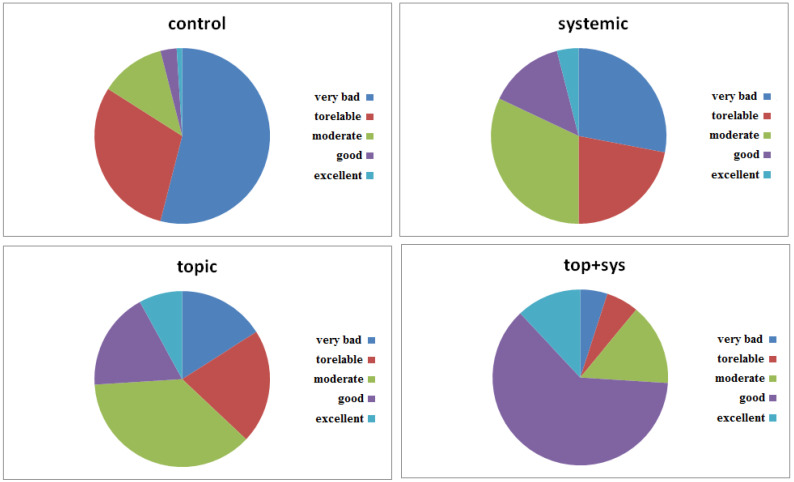
QL tests obtained by collecting the experience of patients subjected to the therapies indicated in the Methods section. Each parameter in the graph corresponds to the percentage of survey responses after therapy. The answers, although subjective and linked to the patient’s sensitivity, can represent the intrinsic value of the well-being achieved by the patient following the therapy.

From Figure 8, which represents the QL trend in patients treated with the experimental protocol mentioned, it can be seen that the PU treatment, to which the patients were subjected, not only showed improvement and the healing of the wound but also an increase in the QL of the patient. The test proposed to patients included several questions aimed at highlighting five fundamental points of the QL scale. In particular, the evaluation of the patient’s general condition, both physical and mental, was required. As can be seen from Figure 8, the percentages of positive outcomes were very high. In particular, in patients treated with the combined therapy (topical+systemic), the “Good” level of satisfaction was 62%, followed by “excellent” at 12% and “moderate” at 15%. The “tolerable” and “very bad” percentages reached 6% and 5%, respectively. In conclusion, it was possible to deduce that the proposed treatment was widely appreciated by a large number of patients. These data, when compared with the results of conventional therapy, highlighted the effectiveness of the application of the biodynamic supplement Citozym in the therapy of pressure ulcers. Conventional treatment highlighted a low level of QL with “very bad” percentages of 54% and “tolerable” percentages of 30%. Furthermore, from Figure 8, it is also possible to deduce that the systemic treatment alone was less effective than the topical treatment.

## 4. Conclusions

The treatment of PU represents a therapeutic challenge due to the wide variety of treatments proposed. The diversity of treatments, the limited amount of valid experimental evidence, and the divergent opinions of healthcare professionals involved in wound care increase therapeutic confusion. The proof of this is that for chronic wounds, such as PUs, and also for diabetic foot ulcers, international guidelines propose diagnostic procedures based mainly on personal experience. The result of this is that the type of topical or systemic wound treatment is often based on the personal opinion of the healthcare professional. This study demonstrated that, within 60 days, it is entirely possible to stimulate the healing process of pressure ulcer wounds, which are difficult to heal, using a biodynamic food supplement (Citozym^®^) applied topically and systemically. All of these elements, in combination, have been found to be directly linked to the wound healing process, even in diabetic patients. Regarding the clinical and laboratory parameters evaluated during the study, no significant differences were found in blood pressure, glycemia, renal function (creatinine), albumin, or pre-albumin levels. To our knowledge, this is the first published study highlighting the utility of this specific blend of enzyme-based bioactive nutrients in wound healing. Furthermore, our sample size was quite large, and the inclusion or exclusion criteria did not significantly affect the generalizability of the data. The strength of our study is such that specific micronutrients from Citozym^®^ were administered in the context of adequate nutritional care, and patients received similar nutritional support to promote new tissue formation. Likewise, the rate of patients who were generally unresponsive to wound care was similar among the four treatment groups and was less than 2%. Regarding the generalizability of the data, less stringent exclusion criteria were allowed due to the age of the patients with a certain degree of comorbidity, although some restrictions were unavoidable. Our findings apply to long-term care residents and patients receiving home care services and, given the aging population, could have a large effect on public health. Other settings, such as intensive care units, were not considered. In our study, adherence to therapy was high because it was integrated with daily care, distributed throughout the day, and not left to the patient’s initiative and ability to undertake it. In conclusion, in malnourished patients with PU receiving nutritional support and guideline-based wound care management, the use of an active biodynamic and enzymatic dietary supplement provides additional wound-healing benefits. The issue of cost-effectiveness should be addressed to support its use. Nutritional intervention should be considered an integral part of pressure ulcer care. The authors thank all healthcare workers and providers for their assistance in recruitment, clinical support, and data collection.

## Figures and Tables

**Table 1:** One-way analysis of variance (ANOVA and Tukey–Kramer method) was performed with Past 4.15. All analyses were considered significant at *p* < 0.05. When comparing the control to the topic, systemic, and topic + systemic groups, there were no significant differences (^ns^).

Treatment	Age (Years)	Height (cm)	Weight (kg)	Body Mass Index	Sex
Control	65.3 ± 9.2	175 ± 11	69.5 ± 10.3	22.8 ± 4.3	Male
Topic	67.2 ± 10.4 ^ns^	175 ± 13 ^ns^	70.5 ± 13.2 ^ns^	23.2 ± 4.7 ^ns^	Male
Systemic	60.0 ± 10.1 ^ns^	175 ± 13 ^ns^	74.4 ± 13.2 ^ns^	24.5 ± 4.9 ^ns^	Male
Topic + Systemic	65.7 ± 11.2 ^ns^	175 ± 15 ^ns^	74.9 ± 14.1 ^ns^	24.9 ± 6.2 ^ns^	Male

**Table 2:** Biometric parameters and presence of diseases/dependencies in each patient included in the control group (*n* = 20). BMI: body mass index.

Age (Years)	Height (cm)	Weight (kg)	BMI	Sex	Diabetes	Smoking	Alcoholic	Hypertensive	Overweight
72	180	60	18.5	male		YES			
70	165	80	29.4	male	YES				YES
65	189	82	23.0	male			YES	YES	
69	165	54	19.8	male	YES				
71	187	75	21.5	male					
62	150	63	28.0	male				YES	YES
58	180	72	22.2	male	YES				
67	175	75	24.5	male					
48	168	82	29.1	male				YES	YES
43	190	77	21.3	male	YES				
72	170	65	22.5	male					
69	180	57	17.6	male			YES	YES	
75	165	66	24.2	male	YES				
70	180	54	16.7	male					
54	176	66	21.3	male					
65	168	87	30.8	male	YES			YES	YES
66	176	68	22.0	male					
75	187	80	22.8	male	YES	YES			
78	190	54	15.0	male					
56	168	72	25.5	male					YES

**Table 3:** Biometric parameters and presence of diseases/dependencies in each patient included in the topic group (*n* = 20). BMI: body mass index.

Age (Years)	Height (cm)	Weight (kg)	BMI	Sex	Diabetes	Smoking	Alcoholic	Hypertensive	Overweight
65	150	59	26.2	male				YES	YES
58	177	75	23.9	male					
78	187	55	15.7	male	YES			YES	
59	158	58	23.2	male					
64	177	48	15.3	male					
65	186	76	22.0	male					
76	190	80	22.2	male			YES		
73	177	65	20.7	male	YES				
75	157	72	29.2	male				YES	YES
77	180	80	24.7	male					
47	160	77	30.1	male					YES
57	159	66	26.1	male	YES				YES
78	200	93	23.3	male					
80	188	74	20.9	male					
77	168	88	31.2	male	YES			YES	YES
72	178	87	27.5	male					YES
54	166	54	19.6	male		YES			
68	186	48	13.9	male	YES			YES	
48	175	76	24.8	male					
73	180	78	24.1	male					

**Table 4:** Biometric parameters and presence of diseases/dependencies in each patient included in the systemic group (*n* = 20). BMI: body mass index.

Age (Years)	Height (cm)	Weight (kg)	BMI	Sex	Diabetes	Smoking	Alcoholic	Hypertensive	Overweight
47	167	77	27.6	male			YES	YES	YES
48	195	70	18.4	male	YES				
49	164	85	31.6	male				YES	YES
60	177	89	28.4	male				YES	YES
51	164	56	20.8	male					
55	185	67	19.6	male	YES				
78	177	65	20.7	male				YES	
70	168	56	19.8	male					
55	190	86	23.8	male	YES			YES	
68	186	95	27.5	male					YES
65	166	58	21.0	male					
58	145	63	30.0	male	YES			YES	YES
46	177	88	28.1	male			YES		YES
60	180	76	23.5	male					
66	165	86	31.6	male	YES				YES
52	170	96	33.2	male				YES	YES
56	178	56	17.7	male	YES				
78	200	75	18.8	male					
75	165	66	24.2	male		YES		YES	
63	180	77	23.8	male					

**Table 5:** Biometric parameters and presence of diseases/dependencies in each patient included in the topic + systemic group (*n* = 20). BMI: body mass index.

Age (Years)	Height (cm)	Weight (kg)	BMI	Sex	Diabetes	Smoking	Alcoholic	Hypertensive	Overweight
67	186	58	16.8	male	YES				
68	177	87	27.8	male			YES	YES	YES
77	188	92	26.0	male					YES
70	154	54	22.8	male	YES				
72	176	74	23.9	male				YES	
67	155	88	36.6	male	YES				YES
58	166	64	23.2	male					
54	186	86	24.9	male					
75	195	71	18.7	male	YES				
70	150	88	39.1	male				YES	YES
72	200	94	23.5	male	YES				
78	154	70	29.5	male					YES
75	188	54	15.3	male	YES				
80	177	73	23.3	male			YES	YES	
48	163	62	23.3	male	YES				
54	186	85	24.6	male					
57	165	88	32.3	male	YES				YES
78	176	90	29.1	male				YES	YES
46	173	54	18.0	male	YES		YES		
47	186	65	18.8	male					

## Data Availability

The original contributions presented in the study are included in the article/Supplementary Material, further inquiries can be directed to the corresponding author.

## Supplementary Materials

The following supporting information can be downloaded at https://www.mdpi.com/article/10.3390/biomedicines12081918/s1. Citozym^®^ Pressure Ulcer Treatment Protocol.
