# Supplementary material for: Therapeutic Potential of a Biodynamic Supplement on Skin Pressure Ulcers: A Randomized Clinical Study
Source: Biomedicines. 2024 Aug 22;12(8):1918. doi: 10.3390/biomedicines12081918 (PMC11351901; doi:10.3390/biomedicines12081918)
Supplement: Supplementary file 1 [file biomedicines-12-01918-s001.zip › biomedicines-3006082-supplementary.pdf]

Attachment "therapeutic treatment"

UNIVERSITÀ DEGLI STUDI DI ROMA "TOR VERGATA"
DIPARTIMENTO DI BIOLOGIA

Citozym® Pressure Ulcer Treatment Protocol

Premises:

1: Always use the classic medical protocol for sterility and cleansing of pressure ulcers (in particular the possible use of antibiotics)
2. Wash the affected area and remove any fragments of necrotic tissue
3. Use a 20% Citozym® solution obtained with sterile and double distilled water for daily washing.
4. At night it is advisable to keep the wound with a medium bandage with gauze soaked in Citozym® in the concentration applied during the day.
5. Check the ulcer after thorough washing and removal of any tissue fragments or exudates. Examine the possible formation of necrotic tissue or signs of infection and inflammation (the possible use of local antibiotics or topical non-corticosteroid anti-inflammatory is permitted). Record any adverse events; infections, allergic reactions, suppuration, inflammation of the surrounding skin, particularly in systemic treatment.

Topical Therapy

1. For 20 days, every 6 hours apply gauze soaked in a 30% Citozym® preparation to the affected area. Then for a further 20 days every 6 hours apply gauze soaked in 40% Citozym®.
2. continue the topical treatment for another 20 days with 70% Citozym® solution.
3. during the night, keep the wounds covered with sterile gauze soaked in the Citozym® solution in the concentration used during the day.

Systemic Therapy

1.Add 10 mL of pure Cytozim® to the patients' diet at breakfast and main meals (30 mL per day). Record any adverse events (nausea, vomiting, dysentery, aerophagia, constipation, etc.). Report any signs of patient suffering to the research manager.

Topical + Systemic Therapy

1.Unify the two therapies described previously.
2.Record any unexpected events and check that the patient follows the healthcare provider's instructions.

Final instructions and preparation for evaluation of patient parameters

The collection of data necessary for the drafting of the experimental protocol is assigned to the researchers responsible for the scientific program. Maximum attention is required when filling out the forms prepared for this operation. All appropriate instrumentation must be examined and calibrated within the necessary times and must be ready at the time of the prospective examination of the patient.

Rome January 20 2023

Prof. Dr. Beninati S.

UNIVERSITÀ DEGLI STUDI DI ROMA "TOR VERGATA"
DIPARTIMENTO DI BIOLOGIA
